# Supplementary material for: Content-rich biological network constructed by mining PubMed abstracts
Source: BMC Bioinformatics. 2004 Oct 8;5:147. doi: 10.1186/1471-2105-5-147 (PMC528731; doi:10.1186/1471-2105-5-147)
Supplement: Additional File 5 — The original Chilibot query results of the term "long-term potentiation (LTP)" and 22 other terms, limiting the latest references analyzed to the years 1990, 1995, 2000, and 2004. [file 1471-2105-5-147-S5.bz2 › chilibotAdditionalFile5/ltp1995/html/CAMKII_ACTIN.html]

 


 **CAMKII** and **ACTIN** 
  
Found 5 abstracts in PubMed,  **5 abstracts were retrieved and analyzed**.  


---

 Search Google  |
 PDF files only 
|  EDU domain only 

---

- J Neurobiol, 1994   **Kinase requirement for retinal growth cone motility.** .
  Since cytoplasmic Ca2ion levels are reported to regulate neurite elongation, we tested whether calcium activated kinases might be necessary for growth cone motility and neurite elongation in explant cultures of goldfish retina.
  Kinase inhibitors and activators were locally applied by micropipette to retinal growth cones and the responses were observed via phase contrast videomicroscopy.
  In some cases, growth rates were also quantified over several hours after general application in the medium.
  The selective inhibitors of protein kinase C, calphostin C 0.1 1 microM and chelerythrin up to 50 microM , caused no obvious changes in growth cones or neurite elongation, and activators of PKC phorbols, arachidonic acid, and diacylglycerol also were generally without effects, although phorbols slowed the growth rate.
  Inhibitors of protein kinase A and tyrosine kinases also produced no obvious effects.
  The calmodulin antagonists, calmidazolium 0.1 microM , trifluoperazine 100 microM , and CGS9343B 50 microM caused a reversible growth cone arrest with loss of filopodia and lamellipodia.
  The growth cone became a club shaped swelling which sometimes moved a short distance back the shaft, leaving evacuated filaments at points of strong filopodial attachments.
  A similar reversible growth cone arrest occurred with the general kinase inhibitors H7 at 200 but not at 100 microM, and staurosporine at 100 but not 10 nM, suggesting possible involvement of a calmodulin dependent kinase camK rather than PKC.
  The selective inhibitor of **camKII**, KN 62 tested up to 50 microM , produced no effects, but the specific myosin light chain kinase MLCK inhibitors ML 7 3 5 microM and ML 9 5 10 microM reversibly reproduced the effect, suggesting that MLCK rather than **camKII** is necessary for growth cone motility.
  The MLCK inhibitors effects both on growth cone morphology and on F **actin** filaments rhodamine phalloidin staining were similar to those caused by cytochalasin D 5 microM , and are discussed in light of findings that inhibiting MLCK disrupts **actin** filaments in astrocytes and fibroblasts.

  - J Neurochem, 1993   **Evaluation of RNAs present in synaptodendrosomes dendritic, glial, and neuronal cell body contribution.** .
    Synaptodendrosomes are subcellular fractions that contain pinched off axon terminals and dendrites.
    These fractions are a potential source of RNAs that are localized in dendrites.
    However, these fractions may also contain RNAs that are seen in situ in neuronal cell bodies and glia.
    To evaluate whether synaptodendrosomes could be used as a source of dendritic RNA, we studied the RNA content of this fraction as compared with RNA isolated from total forebrain and a cell body enriched fraction.
    RNA was analyzed by gel electrophoresis, oligo dT chromatography, and northern blot hybridization.
    RNA from synaptodendrosomes contained a greater proportion of low molecular weight nonpolyadenylated RNAs.
    RNAs known to be present in dendrites mRNA for the alpha subunit of the calcium calmodulin dependent protein kinase II **[CAMKII]** and the polymerase III transcript BC1 were detected in synaptodendrosomes.
    RNAs that are restricted to neuronal and glial cell bodies mRNAs for the 68 kDa neurofilament protein, 43 kDa growth associated protein, beta tubulin, and beta **actin** were present only at low levels.
    However, the mRNA for glial fibrillary acidic protein seen in situ in glial cell bodies and processes was present at high levels in the synaptodendrosomes.
    These results support and extend previous studies indicating that a limited subset of mRNAs is present in neuronal and astrocyte processes and reveal that both of these types of mRNAs are present in synaptodendrosomes.
    Thus, synaptodendrosomes may be useful as a source of dendritic RNAs, but it will be necessary to develop strategies to subtract mRNAs present in astroglial processes.

    - Biochemistry, 1990   **Nucleotide sequence and expression of a cDNA encoding chick brain **actin** depolymerizing factor.** .
      Chick brain **actin** depolymerizing factor ADF is a 19 kDa protein that severs **actin** filaments and binds **actin** monomers.
      We have obtained a cDNA encoding ADF by screening a chick embryo lambda gt11 cDNA library with both a rabbit anti ADF antiserum and two oligonucleotide probes.
      Several non full length clones of 636 bases and one full length clone of 1886 bases were isolated and sequenced.
      The full length cDNA encodes a protein of 165 amino acids with a calculated molecular weight of 1 20.
      The deduced amino acid sequence shows 73% identity with the porcine brain **actin** binding protein cofilin.
      The coding region of the ADF cDNA has been placed in an expression vector, and the resulting protein shows immunoreactivity with an anti ADF antiserum but not with an anti cofilin antibody.
      The expressed ADF has been purified and has an **actin** depolymerizing activity identical with that of brain ADF.
      Like cofilin, ADF contains a sequence similar to the nuclear transport signal sequence of the SV40 large T antigen and a calcium calmodulin dependent protein kinase II **[CAMKII]** phosphorylation consensus sequence.
      Northern blots of both embryonic chick brain and muscle RNA revealed two ADF mRNAs of length 2.1 and 0.9 kilobases.
      Southern blots suggest that the ADF gene is present in a single copy within the chicken genome.
      ADF contains regions of homology with other **actin** binding proteins including tropomyosin, gelsolin, and depactin.

      - J Neurosci, 1989   **Phosphorylation dependent inhibition by synapsin I of organelle movement in squid axoplasm.** .
        Synapsin I, a neuron specific, synaptic vesicle associated phosphoprotein, is thought to play an important role in synaptic vesicle function.
        Recent microinjection studies have shown that synapsin I inhibits neurotransmitter release at the squid giant synapse.
        the inhibitory effect is abolished by phosphorylation of the synapsin I molecule Llinas et al.
        , 1985 .
        We have considered the possibility that synapsin I might modulate release by regulating the ability of synaptic vesicles to move to, or fuse with, the plasma membrane.
        Since it is not yet possible to examine these mechanisms in the intact nerve terminal, we have used video enhanced microscopy to study synaptic vesicle mobility in axoplasm extruded from the squid giant axon.
        We report here that the dephosphorylated form of synapsin I inhibits organelle movement along microtubules within the interior of extruded axoplasm.
        phosphorylation of synapsin I on sites 2 and 3 by calcium calmodulin dependent protein kinase II **[CAMKII]** removes this inhibitory effect.
        Phosphorylation of synapsin I on site 1 by the catalytic subunit of cAMP dependent protein kinase only partially reduces the inhibitory effect.
        In contrast to the inhibition of movement along microtubules seen within the interior of the axoplasm, movement along isolated microtubules protruding from the edges of the axoplasm is unaffected by dephospho synapsin I, despite the fact that the synapsin I concentration is higher there.
        Thus, synapsin I does not appear to inhibit the fast axonal transport mechanism itself.
        Rather, these results are consistent with the possibility that dephospho synapsin I acts by a crosslinking mechanism involving some component s of the cytoskeleton, such as F **actin**, to create a dense network that restricts organelle movement.
        The relevance of the present observations to regulation of neurotransmitter release is discussed.

        - NatureNature,   **Synapsin I bundles F **actin** in a phosphorylation dependent manner.** .
          Synapsin I is a neuron specific phosphoprotein localized to the cytoplasmic surface of synaptic vesicles.
          This phosphoprotein is a major substrate for cyclic AMP dependent and calcium calmodulin dependent protein kinases.
          Its state of phosphorylation can be altered both in vivo and in vitro by a variety of physiological and pharmacological manipulations known to affect synaptic function.
          Recent direct evidence suggests that it may be involved in the regulation of neurotransmitter release from the nerve terminal.
          In the nerve terminal, synaptic vesicles are embedded in a cytoskeletal network, consisting in part of **actin**.
          We report here the ability of the dephospho form of synapsin I to bundle F **actin**.
          This bundling activity is reduced when synapsin I is phosphorylated by cAMP dependent protein kinase and virtually abolished when it is phosphorylated by calcium calmodulin dependent protein kinase II **[CAMKII]** or by both kinases.
          These results, demonstrating an interaction of synapsin I with **actin** in vitro, support the possibility that synapsin I is involved in clustering of synaptic vesicles at the presynaptic terminal.
          the phosphorylation of synapsin I may be involved in regulating the translocation of synaptic vesicles to their sites of release.
